# How Much Emotional Intelligence Effect on Health Centers Performance? A Structural Equation Modeling Approach

**Published:** 2019-08-19

**Authors:** Hojat Gharaee, Razieh Jahanian, Yadollah Hamidi, Ali Reza Soltanian, Ahmad Heidari Pahlavian, Hossein Erfani

**Affiliations:** ^1^Health Center of Hamadan City, Hamadan University of Medical Sciences, Hamadan, Iran; ^2^Vice Chancellor for Health, Hamadan University of Medical Sciences, Hamadan, Iran; ^3^Department of Health Management and Economics, School of Public Health, Hamadan University of Medical Sciences, Hamadan, Iran; ^4^Research Center for Health Sciences, Hamadan University of Medical Sciences, Hamadan, Iran; ^5^Modeling of Noncommunicable Diseases Research Center, School of Public Health, Hamadan University of Medical Sciences, Hamadan, Iran; ^6^Department of Psychology, Member of the Behavioral Disorders and Substance Abuse Research Center, Hamadan University of Medical Sciences, Hamadan, Iran; ^7^Center for Communicable Disease Control, Ministry of Health and Medical Education, Tehran, Iran

**Keywords:** Emotional intelligence, Delivery of health care, Work performance

## Abstract

**Background:** Emotional intelligence (EI) is very important factor to guide managers in a way that leads to access long and short-term organizational goals. The aim of this study was to detect how much emotional intelligence effect on health centers performance.

**Study design:** This is a correlational/analytical study and due to providing some operational strategies for technical health managers and policy makers, it is an applied study.

**Methods:** This study was conducted in 2016 and the population was technical health managers of city health centers in Hamadan Province, western Iran selected by census method. To assess the performance, applied the score that managers have gained in health deputy monitoring. EI measured by Shiring Siberia questionnaire. Data analysis implemented by SPSS software using multiple linear regression, Spearman correlation and Structure Equation Model (SEM).

**Results:** Emotional intelligence of managers has a direct and significant impact on their performance (*P*=0.001), linear regression shows that an increment in the emotional intelligence score of managers, 0.718 units will be added to the performance score. Using multiple regression analysis, the severity of each dimension effect on performance were evaluated which awareness has greatest impact (*P*=0.001, B=0.017) and self-control has the weakest impact (*P*=0.014, B=-0.08) on performance.

**Conclusion:** The level of Emotional intelligence and its aspects has a significant effect on the manager's performance. This fact demonstrates need to high attention of health top managers and decision-makers to enhance health managers EI skills.

## Introduction

The implementation of primary health care needs a comprehensive and effective health care system^1^. Iran’s health system has organized at three levels: nation, province and district. At the district level, one of the sub-units of district Health Network is the City Health Center (CHC), which is the last decision-making authority in the field of management services related to the headquarter units and allocation of resources^1^. Improving healthcare performance is a prominent universal program and represents a significant part of improving public health across the world^2^. One of the most significant features seems to be related to effectiveness, success and performance of health managers in the health organizations is the Emotional intelligence (EI). EI can affect the improving logical actions, such as judgment, decision-making and priority setting, which are important issues in the success and improving performance of manager^3-5^. EI includes a set of abilities, talents and non-cognitive skills that improve individual capability to successfully manage environmental demands and requirements^6-8^.

Human relations, perceiving and feeling of others, self-control, control of the momentary demands, kindness to others, and apply the emotions in thinking and understanding, are subjects of emotional intelligence^9^. There is an overall agreement that EI plays an important role in work settings to regulate and strengthen emotions, attitudes and behaviors among employees and managers^10^.

Emotional intelligence is a skill to identify the meaning of emotions and relationship between them and the ability to monitor their own feelings and others^11^. Goleman introduced the concept of EI, which includes managing with stress, demands and forces created by organizational changes^12^. Emotional Intelligence define as a kind of social intelligence, which contains the skill to perceive self and others emotions, differentiate between them and handle information to lead other`s behavior^13^. However, this is not a unified concept; it has two main forms: EI as theoretical concept at work environment or as a form of intelligence or personality trait^14^.

Goleman, therefore, emphasizes the importance of EI in the workplace and believes that not only managers, but also everyone who workings in the organization requires EI ^15, 16^. Job performance refers to "the value and quantity of activities completed by an individual or group at the workplace. The behavior of employees at work should help the organization's purpose ^17, 18^. Therefore, performance must be defined as the job outcomes, since it has a strong relationship with organization strategic objectives, client satisfaction and economic results^19^.

Since the health system in each country plays an effective role in the development of the country^20^ and health organizations without experienced managers cannot be effective and efficient, therefore investing in empowering managers and promoting their communication skills and emotional intelligence leads to provide more effective and efficient health services in the community.

Given the importance of primary health care, the effect of emotional intelligence on job performance of CHC’s managers has been examined in this study. The main question was how much emotional intelligence effect on job performance of CHC’s managers and which components of emotional intelligence was the best predictor of performance?

## Methods

This was a correlational and analytical study conducted in 2016. The research community was the technical health managers of the nine CHCs covered by Hamadan University of Medical Sciences in west of Iran. In this research, no sampling was performed and all participants were selected by census. These include all health managers working in the headquarters of district health networks and CHC of the nine cities covered by Health Center of Hamadan Province. All 108 managers were selected.

We used the Siberia Shering Emotional Intelligence Questionnaire ^21^ to collect data. The Shering emotional intelligence questionnaire consists of 33 questions that evaluate five components of emotional intelligence. These components are self-awareness, self-control, self- motivation, empathy or social consciousness and social skills that respectively include 8, 7, 7, 6 and 5 questions. Each respondent received six separate grades, 5 of related to each dimensions, and one is overall score. Responses are graded according to Likert scale, ranging from 1 to 5. In a study on librarianship and information professionals and psychologists, the validity of the Shering questionnaire has confirmed. Moreover, the reliability of the questionnaire was 0.92, using Cronbach's alpha ^21^. The level of emotional intelligence and its dimensions has measured through questionnaires answered by the participants themselves. The maximum score of Emotional Intelligence Questionnaire was 165 and the minimum score was 33. If the emotional intelligence’ total score and the score of the dimensions was below 50% of the maximum score was considered poor, between (50.1%) to (75%) intermediate, and above 75% was considered favorable.

Due to the busy work of the managers, researchers referred to the CHC on the day specified by the managers on phone call. After giving the necessary clarifications and responding to the questions of the contributors about the purpose of the study and how to use the results, participants’ written consent received. Then the questionnaire was completed by the contributors, in the presence of the researcher to response the likely questions of the participants.

The performance of the technical health managers of the CHCs is assessing twice a year (every six months) by the health center of province. To determine the level of job performance, the score of managers' performance has used. In this study, the criteria to determine the managers’ job performance were the results of second six months of 2016. The performance scores were judged according to the classification of the province’ health center, which divided the performance into three; poor, intermediate and desirable levels. To collect performance' data, the researchers, with prior coordination, went to the province' health center, and a pre-designed form, for collecting performance data of all CHCs' managers, was completed by the relevant technical units of province health center.

Inclusion and exclusion criteria included employment as the current unit manager for at least six last months and the ability and willingness to participate in the study.

Data were analyzed using SPSS ver.16 software (Chicago, IL, USA). In addition to using frequency distribution tables, Pearson correlation test was used to determine the relationship between emotional intelligence dimensions and performance of managers. Structure equation model (ESM) has used to make latent variable (e.g., emotional intelligence and its subscales) by statistical software AMOS 23. Linear regression model was used to model job performance (i.e., dependent variable) and five subscales of emotional intelligence (i.e., independent variables).

## Ethical considerations

Ethical permission was obtained from Hamadan University of Medical Sciences. A formal letter of cooperation was written to the Management Health Centers. Participants were informed about the purpose, advantage, the confidentiality of the information, and the voluntary nature of participation. The data were collected upon receipt of written consent.

## Results

Findings are presented in two parts of descriptive findings and findings related to hypotheses.

### Descriptive findings

Since at the time of data collection, 7 of the technical health units of the CHCs did not have manager, and 3 of the managers were appointed less than one month as director of the unit, they were excluded from the study. Finally, total participants were 98 that 51 (52%) of them were male and 47 (48%) were female (Table 1). The mean ±SD of participant's age was 41.59±6.47 yr that bounded 33 to 46 years.

**Table 1:** Frequently distribution of participants divided by Marriage and Education

**Variables**	**Male**	**Female**	**Total**
Marital status			
Single	0	12	12
Married	5	35	86
Education			
Diploma	5	0	5
Associate Degree	3	7	10
Bachelor of science	31	30	61
Master of science	4	3	7
PhD	8	7	15

Table 2 shows mean and standard deviation of emotional intelligence dimensions and job performance in nine cities. The overall mean of performance score was 3.59 out of 5. The highest level of performance was 4 and the lowest was 3.11, respectively. Mean of emotional intelligence’ total score was 58.52. Meanwhile, the score of components was, for example, self-motivation (73.55), self-awareness (40.03), self -control (69.3) respectively.

**Table 2:** Mean and standard deviation of emotional intelligence dimensions in nine cities

**Index**	**Self-motivation**	**Self- awareness**	**Self-control**	**Social consciousness**	**Social skills**	**Total**	**Performance**
Mean	73.55	40.03	69.3	73.5	76.28	58.52	3.59
SD	8.15	3.76	10.79	9.98	11.61	6.06	0.97

### The findings of the hypotheses/research questions

Table 3 shows that there was a significant relationship between self-awareness, self-control, self-motivation and sympathy, dimensions of emotional intelligence and manager`s performance (*P***<**0.005). Thus these hypotheses of study are confirmed. On the other hand, there was no significant relationship between social skills dimension of emotional intelligence and manager`s performance (*P*=0.051). In addition, there was a significant relationship between overall emotional intelligence score and manager`s performance (*P***=**0.001).

**Table 3:** Pearson's correlation coefficient for the relationship between emotional intelligence and performance

**Variables**	**Log (performance)**	**Self-motivation**	**Sympathy**	**Self-awareness**	**Self-control**	**Social skills**
Log (performance)	1.000					
Self-motivation	0.396	1.000				
Sympathy	0.372	0.122	1.000			
Self-awareness	0.372	0.396	0.396	1.000		
Self-control	0.310	0.398	0.396	0.396	1.000	
Social skills	-0.638	0.396	0.396	0.396	0.396	1.000

Linear relationship between emotional intelligence (total score) and performance of managers was examined that shows the significant linear relationship between emotional intelligence and performance (*P*<0.001).

**Figure 1:**
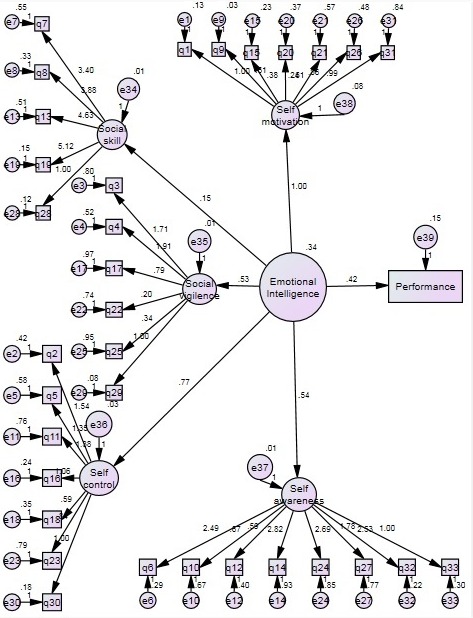
Structure equation diagram for latent emotional intelligence and its latent sub scales

Structure Equation Model (SEM) was used to make latent emotional intelligence and five its sub-scales (i.e., social skill, self-motivation, social vigilance, self-control and self-awareness). Structure equation analysis confirms goodness of fit between emotional intelligence and its sub-scales (RMSE=0.038, GFI=0.87, AGFI=0.89).Structure equation diagram is shown in Figure 1.

Table 4 shows multiple linear regression model (using leading stepwise method) that all dimensions of emotional intelligence except social skills (*P*=0.051) predict the job performance. On the other hand, self-awareness was the strongest variable to predict performance.

**Table 4:** Multiple linear regression model between the components of emotional intelligence and performance by using multiple linear regression (using the leading stepwise method)

**Predictor variables**	**SE**	**B**	**T**	***P*** **value**
Constant coefficient	0.148	-	- 2.958	0.004
Self-motivation	0.004	0.365	4.017	0.001
Sympathy (Social consciousness)	0.003	0.376	4.427	0.001
Self-awareness	0.004	0.341	3.833	0.001
Self-control	0.003	0.237	- 2.494	0.014

log (Performance score) = - 0.437 + 0.014 (Self-motivation) + 0.012 (Sympathy) + 0.017 (Self-awareness) – 0.08 (Self-control)

Finally, linear regression between performance and total scores of emotional intelligence (i.e., a linear combination on five dimensions of emotional intelligence) shows that per an increment in the emotional intelligence score of managers, 0.718 units will be added to the performance score (log performance) = -0.652+0.321 (emotional intelligence), *P*=0.012).

## Discussion

Totally, 108 questionnaires were distributed of which 98 questionnaires were completed and returned (91%). There is a significant correlation among self-awareness, self-control, self-motivation and sympathy dimensions of emotional intelligence with health manager`s performance. Moreover, the results of present study revealed that there is significant relationship between emotional intelligence overall score and health manager`s performance, that means by improving emotional intelligence in terms of these dimensions, the performance of managers improves. In fact, knowing and understanding emotions and recognizing its contribution to organizations and individuals can effective in decision-making and appropriate more rational behaviors of managers and improve performance.

While there was an important correlation between intrapersonal dimension (sympathy and social skills) of emotional intelligence and job performance, there was no relationship with other subscales^9^. There was significant relationship between the self-awareness, self-control and self-motivation and job performance. Moreover, the results of this study indicated that the relationship between Empathy and social skills and job performance was not confirmed^11^. On the other hand, emotional intelligence dimensions empathy, self-motivation and social skills had positively correlated with the performance of managers, but self-control and self-awareness components did not have a significant relationship with the performance of managers^22^. Therefore, emotional intelligence and its all dimensions had a positive and significant effect on the performance of the staff. Any increase in each dimension increases the performance of the staff ^23^. Moreover, this result was approved by Fujino et al study^12^.

In fact, knowing and understanding own’ emotions and identifying its effect on organizations and individuals can affect decision-making and proper and more logical behaviors of managers and develops their job performance. Since this result was not confirmed with some studies ^19, 22^, the impact of this dimension on performance needed to more examination in upcoming studies.

Regression between performance and emotional intelligence total scores (i.e., a linear combination on five dimensions of emotional intelligence) showed that an increase in the EI score, 0.718 units will be added to the performance score. This means that by improving EI, the performance of managers improves. This result is confirmed by many studies ^2, 10, 19, 24-27^. Furthermore, this hypothesis has been confirmed by the meta-analysis study^28^. In another study, this result was not approved^29^.

Successful health managers use the abilities of emotional intelligence very harmoniously, which we have recognized them as the key to high executive performance of a manager. Managers, who have high EI, have special skills in executing their jobs, show better performance and can make positive attitudes and intuition between their personnel ^24, 26, 27^.

Health managers by understanding emotions of others, expressing positive and empathic feelings and as well as adjust their emotions and control them would create more effective relations with others and, therefore, better acceptance and as a result, will be able to have better interpersonal relations (one of the elements in evaluating job performance). This positive control has excessive and favorable impression on the others evaluation^27^. Above hypothesis was not confirmed by some study ^14, 18, 30^, where, higher emotional intelligence did not lead to better performance of managers necessarily.

Multiple linear regression analysis (stepwise method) showed that all aspects of emotional intelligence (except social skills) have a significant effect on the performance of participants. Self-awareness and self-control have been the strongest and weakest components to predict participants’ job performance scores. In a study, between all dimensions of emotional intelligence, self-motivation had the highest correlation with the managers’ performance ^31^. Training and developing managerial skills especially human and social skills in administrators and managers of CHCs can decrease job stress and enhance effective performance^32^. Social skills and self-motivation were the strongest and weakest dimensions to predict the performance scores respectively ^31^.

## Conclusion

The most important result of this research is to reveal positive correlation between emotional intelligence and its sub-scales with health managers' job performance. In fact increase in the emotional intelligence score of managers, 0.718 units will be added to the performance score. These results reveal the weakness and strengths of technical health managers of CHC in the field of emotional intelligence. Moreover, this study could be an introduction to more comprehensive research with different samples in various departments of Hamadan University of Medical Sciences. The results of such studies could be the basis for health managers’ strengthening programs. In addition, using these results the positive points and weaknesses of monitoring and evaluation systems in CHC can be known and improved.

## Acknowledgements

This study has been adapted from a research project at Hamadan University of Medical Sciences. Thereby appreciate all contributed in this research, particularly health managers who participated in the study.

## Conflict of interest

The authors declare that they have no conflicts of interest.

## Funding

The study was funded by the Vice-chancellor for Research and Technology, Hamadan University of Medical Sciences (No. 930321314).

## Highlights

- Emotional Intelligence (EI) relates to organizational performance and manager's effectiveness.
- Self-awareness has the greatest and self-control has the weakest impact on health managers' performance.
- Increase in EI score of health managers can enhance the performance score
- EI training and education may be helpful to increase managers EI score.
